# The canonical eIF4E isoform of *C. elegans* regulates growth, embryogenesis, and germline sex-determination

**DOI:** 10.1242/bio.011585

**Published:** 2015-05-15

**Authors:** Richard S. Mangio, SarahBeth Votra, David Pruyne

**Affiliations:** 1 Department of Biochemistry and Molecular Biology, SUNY Upstate Medical University, Syracuse, NY 13210, USA; 2 Department of Cell and Developmental Biology, SUNY Upstate Medical University, Syracuse, NY 13210, USA

**Keywords:** *C. elegans*, EIF4E, Germline, Sex-determination, Spermatogenesis, Oogenesis

## Abstract

eIF4E plays a conserved role in initiating protein synthesis, but with multiple eIF4E isoforms present in many organisms, these proteins also adopt specialized functions. Previous RNAi studies showed that *ife-3*, encoding the sole canonical eIF4E isoform of *Caenorhabditis elegans*, is essential for viability. Using *ife-3* gene mutations, we show here that it is maternal *ife-3* function that is essential for embryogenesis, but *ife-3* null progeny of heterozygous animals are viable. We find that zygotic *ife-3* function promotes body growth and regulates germline development in hermaphrodite worms. Specifically, the normal transition from spermatogenesis to oogenesis in the hermaphrodite germline fails in *ife-3* mutants. This failure to switch is reversed by inhibiting expression of the key masculinizing gene, *fem-3*, suggesting *ife-3* resembles a growing number of genes that promote the sperm/oocyte switch by acting genetically as upstream inhibitors of *fem-3*.

## INTRODUCTION

Eukaryotic initiation factor 4-complex (eIF4) recruits mature mRNAs to ribosomes as the first step of translation (Gingras et al., 1999). The factor eIF4E recognizes a methylated guanosine cap at the mRNA 5′ end, aiding recruitment of the complex to the mRNA. Multiple eIF4E homologs are common among organisms (Hernández and Vazquez-Pianzola, 2005), allowing them to adopt specialized functions. The nematode *Caenorhabditis elegans* encodes five eIF4E proteins (IFE-1 to -5) that reflect a diversity partially conserved across the animal kingdom: IFE-3 resembles the canonical eIF4E-1 isoforms of mammals and insects; IFE-4 is a member of the divergent 4E-HP group of eIF4E proteins; and IFE-1, -2, and -5 are closely related isoforms that make a nematode-specific sub-group (Hernández and Vazquez-Pianzola, 2005; Jankowska-Anyszka et al., 1998; Keiper et al., 2000).

Worm eIF4E homologs vary in expression pattern and the effects of their loss. IFE-2 is enriched in the soma, but also functions in the germline. Its loss inhibits general somatic mRNA translation, as well as temperature-dependent translation of germline mRNAs required for meiotic crossover repair (Hansen et al., 2007; Song et al., 2010; Syntichaki et al., 2007). IFE-4 is expressed somatically, and its absence reduces neuronal and egg-laying gene expression, resulting in impaired egg laying (Dinkova et al., 2005). IFE-1, -3, and -5 are germline-enriched (Amiri et al., 2001). No function is known for IFE-5, but IFE-1 loss partially impairs oogenesis, and disrupts spermatogenesis at high temperatures (Amiri et al., 2001; Henderson et al., 2009; Kawasaki et al., 2011). RNA-mediated inhibition (RNAi) studies show IFE-3 is essential for embryogenesis (Keiper et al., 2000). Using *ife-3* gene mutations, we report here additional novel roles for IFE-3 in postembryonic development, particularly in promoting the transition of the hermaphrodite germline from a spermatogenic to an oogenic tissue.

## RESULTS

### Zygotic *ife-3* is not essential for viability, but is important for normal body size

**Fig. 1.**
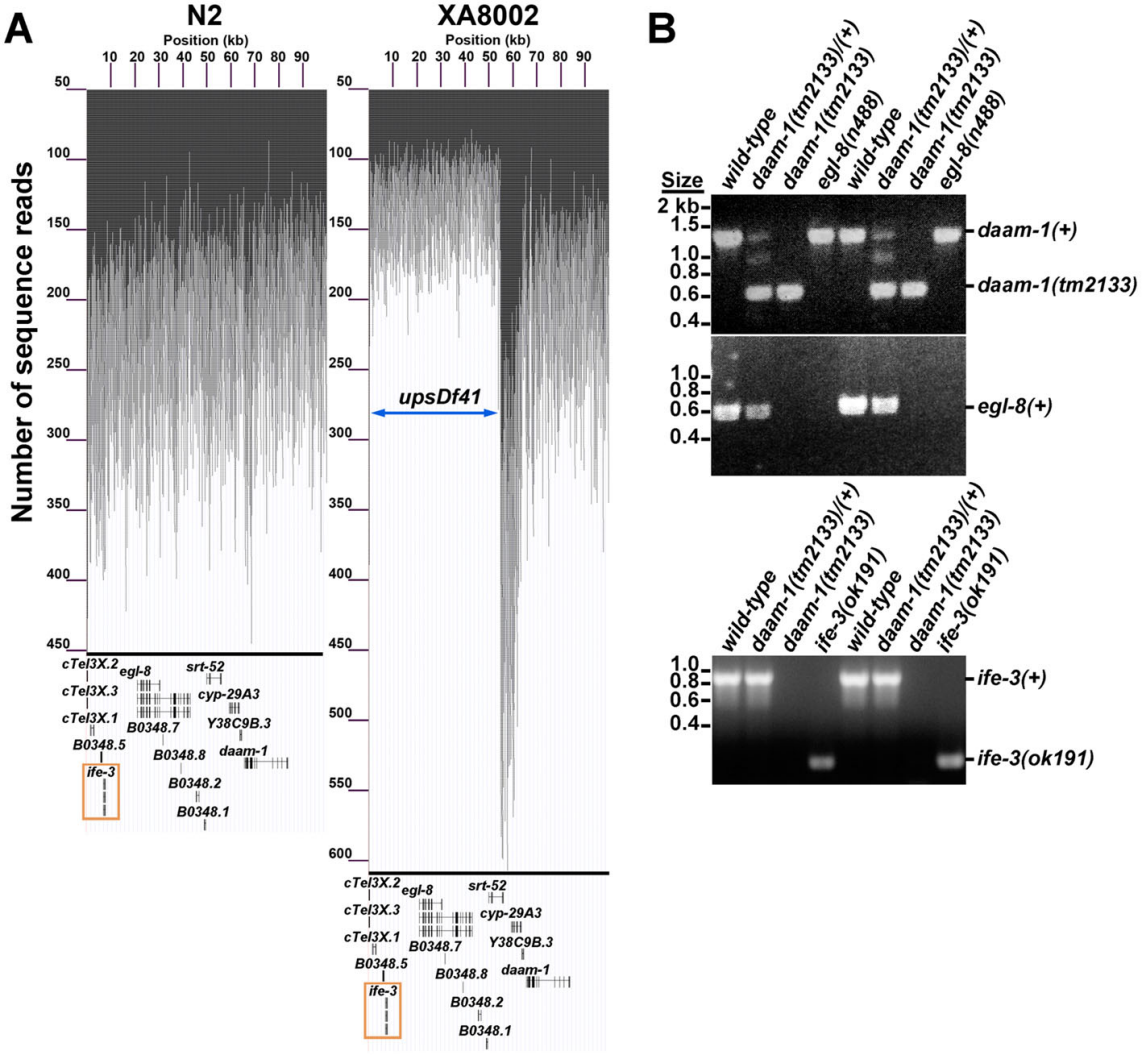
**A large genomic deletion, *upsDf41*, is linked to *daam-1(tm2133)* and eliminates *ife-3*.** (A) The locations of sequence reads obtained by whole genome sequence analysis were plotted against their position on the first 100,000 bp of ChrV for the wild-type strain N2, and the heterozygous *daam-1(tm2133)/nT1* strain XA8002. The number of sequence reads for each position over the first 100 kb of ChrV for N2 (left) vary around a relatively constant average of ∼200–250 reads per position, excluding the telomeric region. The number of sequence reads per position for XA8002 also vary around ∼200–250 reads per position for ChrV positions greater than +70,000, but over the first ∼50 kb of ChrV, the average number of reads per position is ∼100–150, marking the genomic deficiency *upsDf41.* The average number of reads for the neighboring 8 kb is at least 50% greater, indicating the presence of additional copies of that sequence in XA8002. The exon positions of known genes are displayed beneath, including *ife-3* (boxed). (B) PCR of single worms using primers for *daam-1*, *ife-3*, and *egl-8* shows that the 700 bp deletion *tm2133* of *daam-1* is linked to an absence of *ife-3* and *egl-8*. PCR of control animals bearing the 686 bp deletion *(ok191)* of *ife-3*, or the allele *(n488)* lacking one priming site for *egl-8*, demonstrate the specificity of primers used.

The wild-type *C. elegans* hermaphrodite, being able to produce both sperm and oocytes, is self-fertile. In an analysis of worms mutated for formin family genes, we had reported that a deletion allele *tm2133* of the formin gene *daam-1* is linked to recessive hermaphrodite sterility (Mi-Mi et al., 2012). However, *daam-1(+)* transgenes do not restore fertility to homozygous *daam-1(tm2133)* hermaphrodites, and RNAi against *daam-1* does not induce sterility in wild-type hermaphrodites, suggesting an unidentified linked mutation as the cause (R.S.M., unpublished observations; King et al., 2009). To identify such a mutation, we stably balanced *daam-1(tm2133)* against the genomic transposition *nT1[qIs51]* in the heterozygous strain XA8002, and sequenced the genome of this strain. No identified point mutations or small deletions in XA8002 are likely to cause sterility (supplementary material Table S1), but over several regions near *daam-1*, the sequence coverage was decreased approximately 50%, suggesting these regions are deleted from one copy of Chromosome V (ChrV) in XA8002 (Fig. 1A, supplementary material Table S2). The largest putative deletion spans eleven genes (Table 1). Using single-worm PCR, we confirmed that this large deletion, designated here as *upsDf41*, is present on the *daam-1(tm2133)*-containing ChrV homolog in XA8002 (Fig. 1B).

**Table 1.**
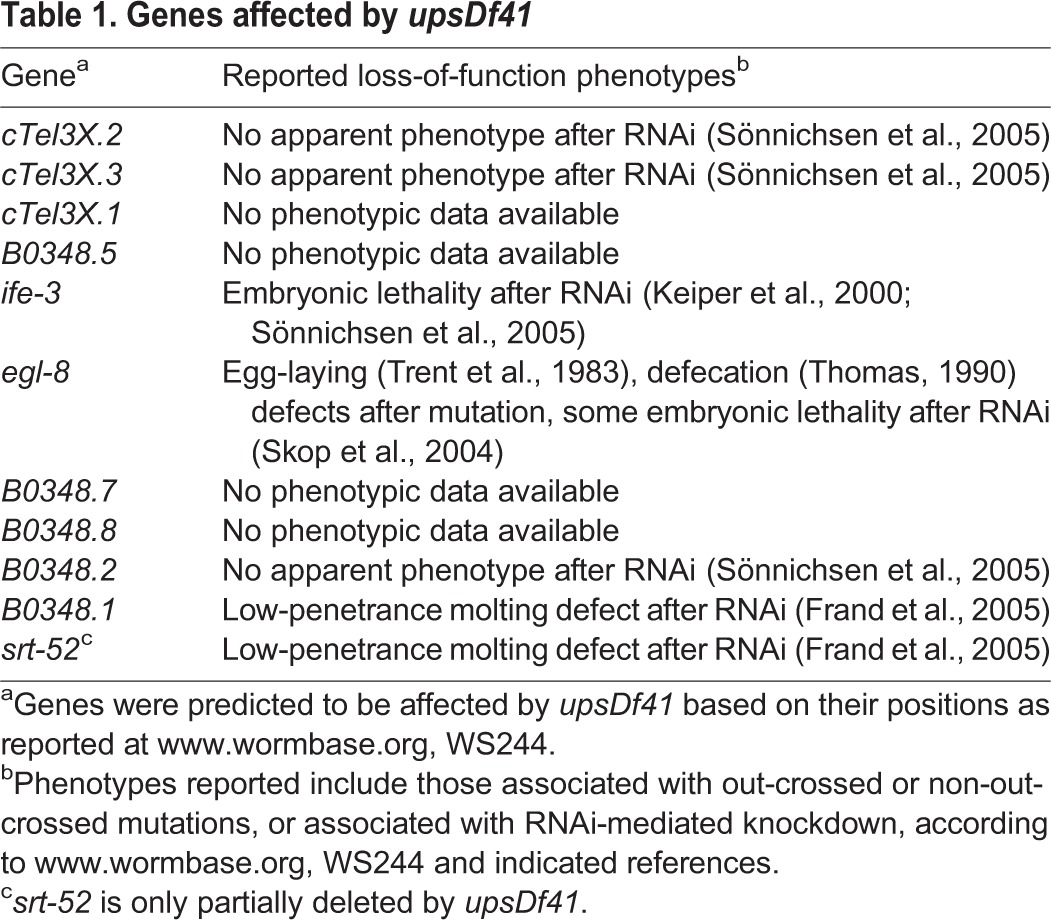
**Genes affected by *upsDf41***

Among the genes disrupted by *upsDf41*, only *ife-3* had been reported to be essential, with RNAi against *ife-3* resulting in 100% embryonic lethality (Keiper et al., 2000). However, we were able to isolate *upsDf41* homozygous worms that completely lacked *ife-3* (Fig. 1B). We also quantitatively tested for association between absence of *ife-3* and embryonic lethality. To avoid the embryonic lethality associated with the genomic transposition *nT1[qIs51]* in XA8002, we first crossed *upsDf41* into a wild-type background. We then isolated individual *upsDf41/+* heterozygous hermaphrodites and wild-type positive control hermaphrodites, and allowed them to lay eggs, and tracked the fate of their progeny. For worms of both genotypes, nearly 100% of their eggs hatched, and nearly 100% of the resultant larvae grew to adulthood (Table 2). Thus, absence of *ife-3* from the zygotic genome does not result in lethality under standard growth conditions.

**Table 2.**
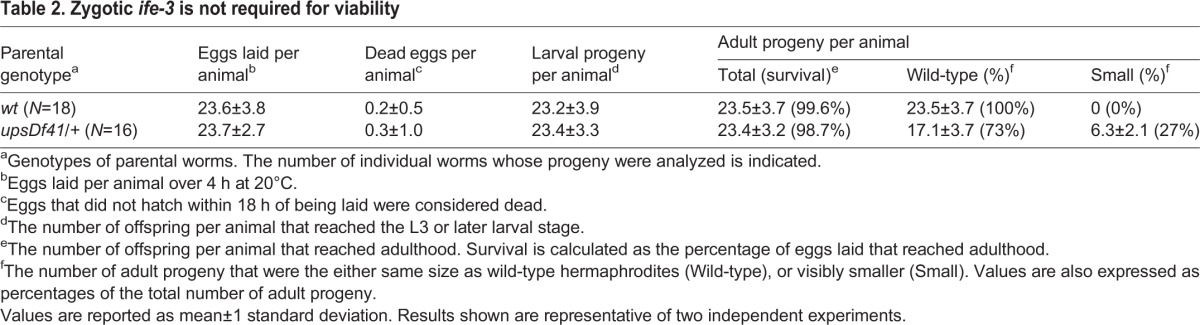
**Zygotic *ife-3* is not required for viability**

**Fig. 2.**
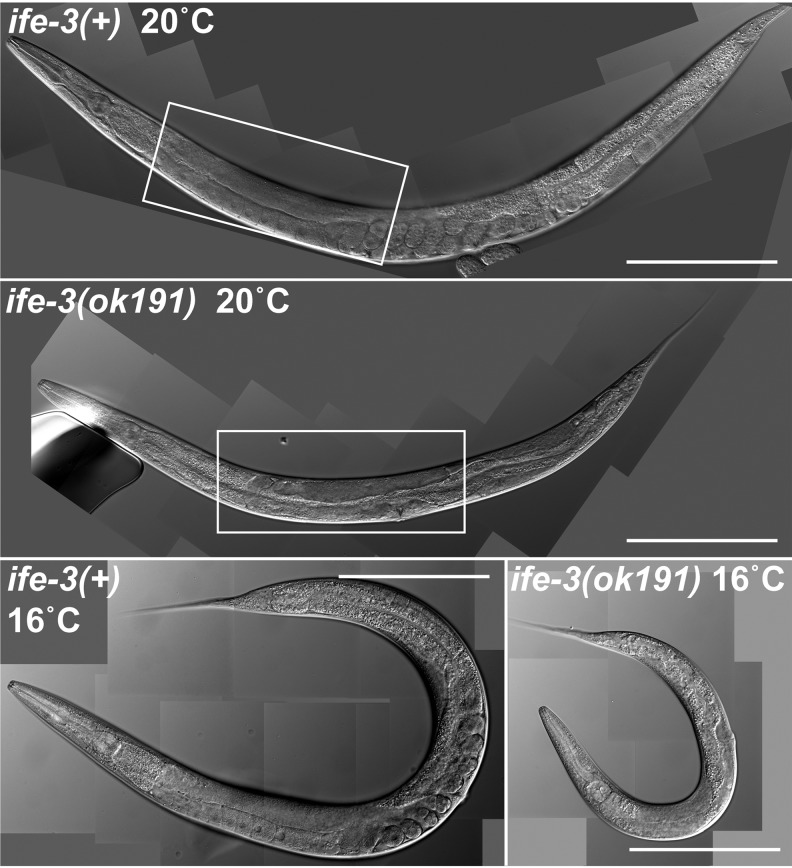
**Growth defect of *ife-3(ok191)* mutants.** Wild-type and *ife-3(ok191)* hermaphrodites were grown to young adulthood at the indicated temperatures. Viewed through DIC microscopy, mutant hermaphrodites are smaller than wild-type animals, particularly at a cooler growth temperature. The boxed regions are reproduced at higher magnification in Fig. 3A. Bars=200 µm.

However, while the adult progeny of wild-type animals appeared wild-type, approximately 27% of the adult progeny of the *upsDf41/+* worms were small, suggesting homozygosity of *upsDf41* or *daam-1(tm2133)* results in poor growth (Table 2). Confirming this, heterozygous XA8002 worms have a normal body size but their *upsDf41 daam-1(tm2133)* homozygous progeny are small (supplementary material Fig. S1A). To test whether absence of *ife-3* contributes to the small size of *upsDf41* homozygotes, we obtained from the Caenorhabditis Genetics Center (University of Minnesota) the worm strain KX10, which is heterozygous for the smaller deletion *ife-3(ok191)* affecting only the immediate upstream sequence and exon 1 of *ife-3* (Wormbase). For ease of analysis, we stably balanced *ife-3(ok191)* with *nT1[qIs51]* in the strain DWP70. As *nT1[qIs51]* encodes a recessive lethal allele and a pharyngeal-expressed GFP, we could unambiguously distinguish GFP-expressing *ife-3(ok191)/+* heterozygous progeny from GFP-lacking *ife-3(ok191)* homozygous progeny. Similar to *upsDf41*, homozygotes for *ife-3(ok191)* are smaller than wild-type or heterozygous animals (Fig. 2). This effect is exaggerated at a cooler growth temperature (Fig. 2). Thus, absence of zygotic *ife-3* is not lethal, but results in poor growth exacerbated by cold.

### Maternal effect lethality of *ife-3* mutants

We observed that hermaphrodites homozygous for *ife-3(ok191)* or for *upsDf41* (isolated as GFP-negative progeny of DWP70 or XA8002, respectively) produce eggs only under particular conditions (see below), but these eggs always terminate development as a mass of cells with no obvious morphogenesis (Table 3 and data not shown). These results indicate that while zygotically-encoded *ife-3* is not essential for embryogenesis, a maternal supply of *ife-3* is required for this process. Notably, this embryonic lethality phenotype matches the effects of RNAi against *ife-3* (Keiper et al., 2000; Sönnichsen et al., 2005).

**Table 3.**
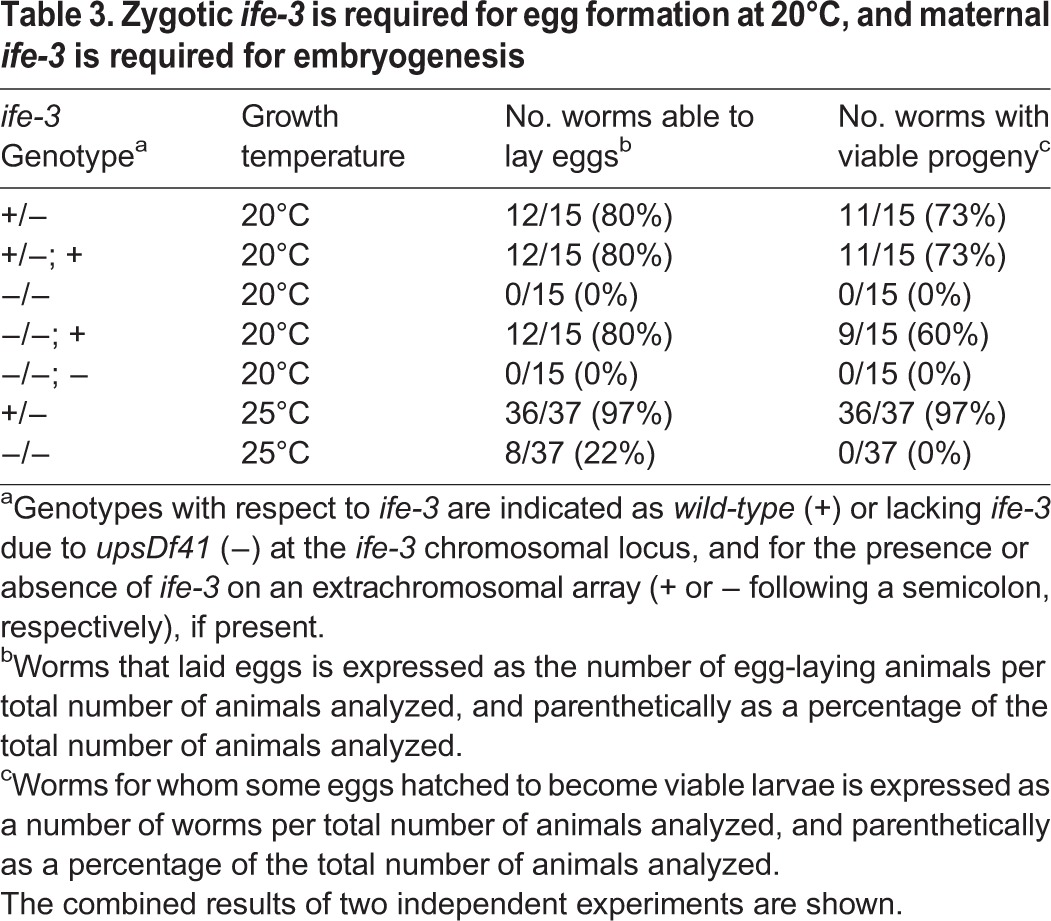
**Zygotic *ife-3* is required for egg formation at 20°C, and maternal *ife-3* is required for embryogenesis**

To test whether the maternal effect lethality associated with *upsDf41* depends on *ife-3*, we microinjected XA8002 worms with wild-type *ife-3* sequence to create the complex extrachromosomal array *upsEx40[ife-3(+)]*. Using co-injected mCherry-expressing markers to follow the inheritance of *upsEx40[ife-3(+)]* among the progeny of injected animals, we found that *upsDf41* homozygous progeny that inherit *upsEx40[ife-3(*+*)]* are fertile, laying eggs that hatch as larvae and grow to adulthood (Table 3). Conversely, *upsDf41* hermaphrodites that inherit a control array lacking *ife-3* remain sterile. Thus, maternal *ife-3*, but not *daam-1* or any other gene affected by *upsDf41* (Table 1), is required for embryogenesis.

### Masculinization of the *ife-3* mutant hermaphrodite germline

While *ife-3(ok191)* or *upsDf41* homozygous hermaphrodites produce inviable eggs under certain circumstances (described below), under normal growth conditions these hermaphrodites do not produce eggs (Table 3). Egg production in the *C. elegans* hermaphrodite begins with the proliferation of germ stem cells in the distal tips of two gonad arms. As these germ cells move in the proximal direction through the arms, they progressively differentiate. During late larval development, germ cells differentiate into spermatids that accumulate in the proximal gonad arms. In early adulthood, spermatogenesis stops and maturing germ cells instead differentiate into oocytes. These much larger cells accumulate in the proximal gonad arms, and push the smaller spermatids into the adjoining spermathecae, where they fully differentiate into sperm (Ward and Carrel, 1979). Throughout the fertile adulthood of hermaphrodites, oocytes are engulfed by the spermatheca during ovulation, where they are fertilized by resident sperm and then pushed into the uterus to commence development as embryos. Fertilized embryos rapidly synthesize eggshells, and after a brief period are laid through the vulva to continue development *ex utero*.

**Fig. 3.**
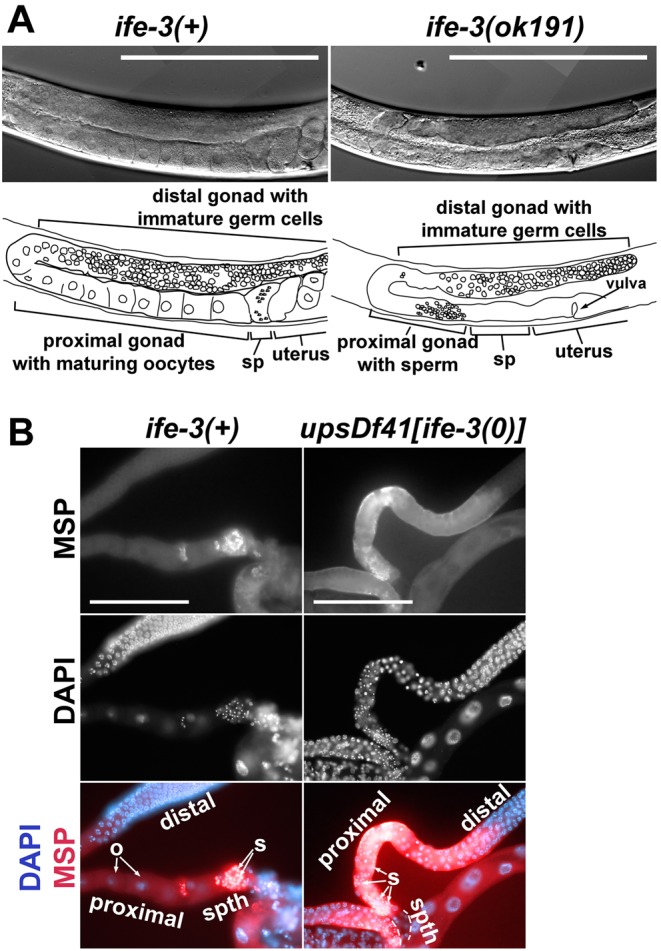
**The gonads of *ife-3* mutant hermaphrodites grown at 20°C lack oocytes and embryos, but contain sperm.** (A) Gonad arms of wild-type and *ife-3(ok191)* hermaphrodites grown to young adulthood at 20°C were viewed through DIC microscopy. In the wild-type gonad, immature germ cells occupy the distal region of a gonad arm, while large oocytes are lined up in the proximal arm, tiny indistinct-appearing sperm occupy the spermatheca (sp), and embryos occupy the uterus. In the *ife-3(ok191)* gonad, immature germ cells occupy the distal arm, but oocytes are absent from the proximal gonad, and the spermatheca and uterus appear empty. Bars=200 µm. (B) Dissected gonad arms of young adult wild-type or *upsDf41* homozygous hermaphrodites (which lack *ife-3*) were stained with DAPI to reveal germline nuclei, and with antibodies specific to MSP, a cytosolic component of sperm and spermatids. In the wild-type gonad arm, large oocytes (o) lacking MSP occupy the proximal region, while small MSP-rich sperm (s) with punctate nuclei are visible in the spermatheca (*spth*). In the *upsDf41* gonad arm, MSP is enriched in small spermatids (s) with punctate nuclei in the proximal region, as well as more diffusely in the gonad, consistent with ongoing sperm production, while the spermatheca is empty of germ cells. In both strains, nuclei of immature germ cells are visible in the distal portion of the gonad arms. Bars=100 µm.

**Fig. 4.**
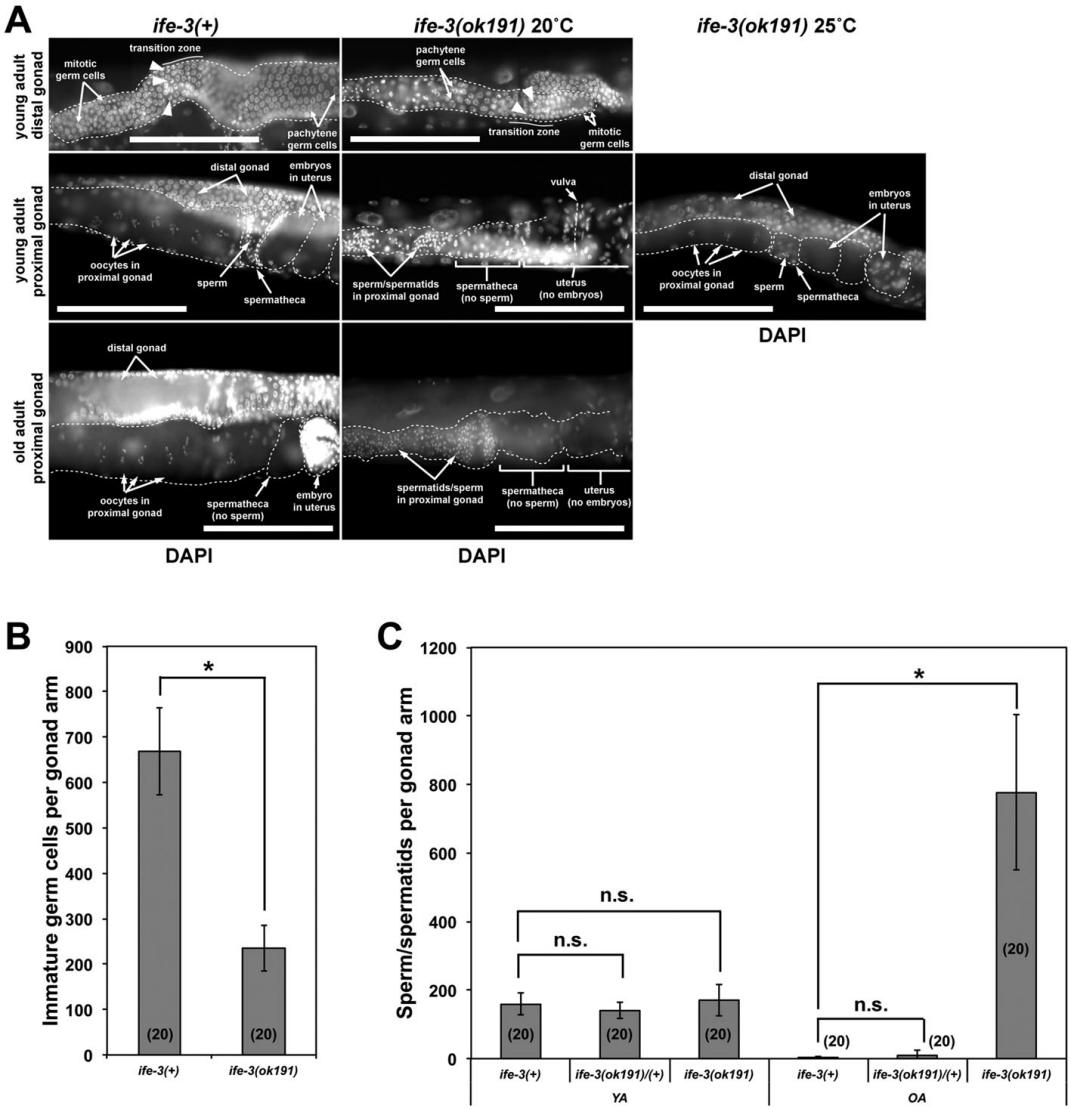
**Spermatogenesis continues through adulthood in *ife-3(ok191)* hermaphrodites.** (A) Wild-type and *ife-3(ok191)* hermaphrodites were stained with DAPI. (Top) Distal gonad arms. In both trains, spherical mitotic germ cell nuclei are near the distal tip, crescent shaped nuclei (arrowheads) of germ cells entering meiosis are present in a neighboring transition zone, and germ cells arrested in pachytene with nuclei containing thread-like chromatin strands occupy the remainder. (Middle) Proximal gonad region of young adults. In the wild-type hermaphrodite, large oocytes with nuclei containing six condensed chromatin bodies are lined up in the proximal arm, while punctate sperm nuclei occupy the spermatheca. In an *ife-3(ok191)* hermaphrodite grown at 20°C, the proximal gonad lacks oocytes but contains sperm/spermatid with punctate nuclei, while the spermatheca and uterus are empty of germ cells. In an *ife-3(ok191)* hermaphrodite grown at 25°C, the proximal gonad resembles that of a wild-type hermaphrodite, with oocytes lined up in the proximal arm, sperm present in the spermatheca, and developing embryos in the uterus. (Bottom) Proximal gonad region of old adults. In the wild-type hermaphrodite, oocytes are lined up in the proximal arm, while sperm have been depleted from the spermatheca. In an *ife-3(ok191)* hermaphrodite grown at 20°C, the proximal gonad contains many spermatids, while the spermatheca and uterus remain empty. Bars=100 µm. (B) The number of immature germ cell nuclei, which includes stem cells, meiotic cells in the transition zone, and pachytene-arrested cells, were counted in the gonad arms of DAPI-stained young adult wild-type and *ife-3(ok191)* homozygous hermaphrodites. The mutant gonad arms contain fewer immature germ cells. Results shown are typical of two independent experiments. (C) The number of sperm/spermatids, identified as punctate DAPI-stained nuclei, declines from young adulthood (YA) to old adulthood (OA) in wild-type and heterozygous hermaphrodites, but increases in *ife-3(ok191)* homozygotes. Results shown are typical of three independent experiments. For B and C, the number of gonad arms analyzed per experiment is indicated parenthetically, and values are expressed as mean±one standard deviation. * indicates *P*<0.0001. n.s. indicates no significant difference.

In young adult wild-type hermaphrodites, developing embryos are easily viewed in the uterus using DIC microscopy, and oocytes are visible as large cells lined up in the proximal gonad (Fig. 3A). In contrast, young adult *ife-3(ok191)* or *upsDf41* homozygous hermaphrodites grown at 16°C or 20°C lack visible oocytes or embryos (Figs 2 and 3A, supplementary material Fig. S1B). Sperm and spermatids can be viewed using DAPI stain of DNA, which reveals their punctate nuclei, or immunofluorescence stain of major sperm protein (MSP), a major cytosolic component of sperm (Kosinski et al., 2005). In young adult wild-type hermaphrodites, sperm are present in the spermatheca (Figs 3B and 4A). In *ife-3(ok191)* or *upsDf41* homozygous hermaphrodites grown at 20°C or lower, sperm/spermatids are also present, but in the proximal gonad rather than the spermatheca, likely as a secondary consequence of the absence of oocytes to push them to their proper place (Figs 3B and 4A, supplementary material Fig. S1B).

DAPI stain also shows that the distal portions of *ife-3(ok191)* and *upsDf41* hermaphrodite gonad arms are small and contain fewer germ cells than in wild-type animals (Fig. 4A,B). However, they contain recognizable germ stem cells in the distal tips, immature germ cells entering meiosis in the transition zone, and germ cells arrested in pachytene, as typical for wild-type animals (Fig. 4A, supplementary material Fig. S1B). Moreover, young adult *ife-3(ok191)* or *upsDf41* homozygous hermaphrodite gonads house a similar number of sperm as young adult wild-type hermaphrodites (Fig. 4C, supplementary material Fig. S1C). However, where wild-type hermaphrodites deplete their sperm over subsequent days through self-fertilization of oocytes, the homozygous mutant hermaphrodites continue to accumulate sperm through adulthood (Fig. 4C, supplementary material Fig. S1C). The number of sperm accumulated in these mutants is much higher than the maximal ∼160 sperm per arm created in wild-type hermaphrodites (Hodgkin, 1988), indicating that germ cells that would normally become oocytes instead differentiate into sperm.

The persistence of spermatogenesis and absence of oogenesis in the *ife-3* mutant germline does not reflect a global larval developmental arrest of the mutants, as *ife-3(ok191)* or *upsDf41* homozygous hermaphrodites complete the final cuticle molt that marks adulthood, and their vulvas open normally at the same time as their heterozygous siblings (data not shown). Also, while their germline is masculinized, the soma of *ife-3* mutants remains that of hermaphrodites, with whip-shaped tails rather than fan-shaped tails like male worms, and with two-armed gonads rather than one-armed gonads like male worms (Fig. 2). Thus, *ife-3* mutants grown at 16°C or 20°C have a masculinization of germline (mog) phenotype.

At the relatively high temperature of 25°C, the mog phenotype is only partially penetrant, with approximately one-fifth of *ife-3(ok191)* or *upsDf41* homozygous hermaphrodites producing oocytes that become fertilized and commence development (Table 3, Fig. 4A). However, as described above, the resultant eggs do not hatch due to maternal effect lethality during embryonic morphogenesis (Table 3). We also rarely observed homozygous mutant hermaphrodites producing oocytes and laying inviable eggs at 20°C, but only when all food had been consumed, suggesting starvation might also bypass the requirement for *ife-3* in the spermatogenesis-to-oogenesis switch. For either permissive condition (25°C or starvation), we only observed egg production when the condition was introduced before the final L4 larval stage, when the spermatogenesis-to-oogenesis transition normally occurs, suggesting the mog phenotype is not reversible once established.

### *ife-3* functions upstream of the masculinizing gene *fem-3*

A key point of control of the spermatogenesis-to-oogenesis transition is regulation of the pro-spermatogenic gene *fem-3* (Ellis and Schedl, 2007). Excess *fem-3* activity results in a mog phenotype similar to *ife-3* mutants, while absence of *fem-3* produces “female” animals that fail to produce sperm, but accumulate large numbers of unfertilized oocytes in their proximal gonad (Hodgkin, 1986; Ahringer and Kimble, 1991; Barton et al., 1987). Many genes that promote the spermatogenesis-to-oogenesis transition act as upstream inhibitors of *fem-3* activity.

**Fig. 5.**
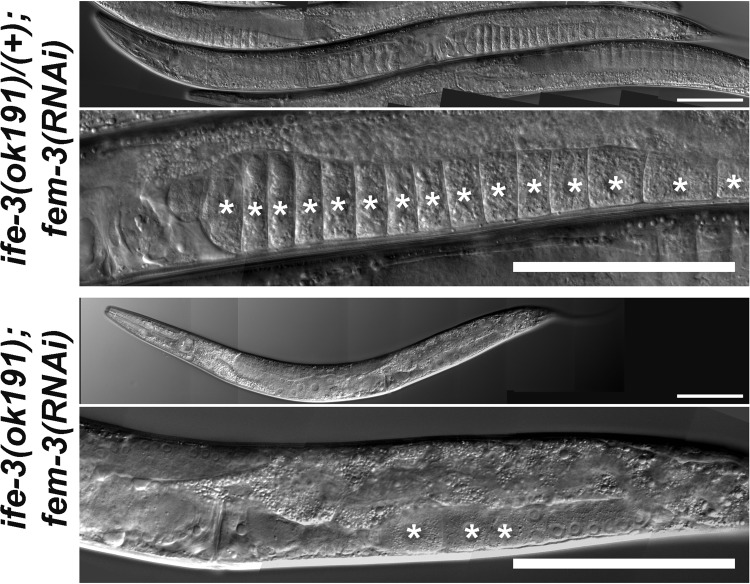
***fem-3* is epistatic to *ife-3*.** Progeny of DWP70 hermaphrodites treated for RNAi against *fem-3* were examined by DIC microscopy. Shown are whole worms and magnified views of one gonad arm. Young adult heterozygous progeny were often female, characterized by a lack of fertilized embryos in their uterus and an excessive number of oocytes (marked by *) in their proximal gonad arms. Young adult *ife-3(ok191)* progeny were also apparently female in that they also lacked embryos and contained oocytes. However, they had only a few small oocytes (*), suggesting a reversal of the mog phenotype by *fem-3(RNAi)*, but not reversal of the poor body or gonad growth phenotypes. Bars=100 µm.

To determine whether *ife-3* might also act as an upstream inhibitor of *fem-3*, we tested whether inhibition of *fem-3* expression bypasses the need for *ife-3* in promoting oogenesis. We induced RNAi against *fem-3* by microinjecting *fem-3*-encoding dsRNA into the gonads of wild-type hermaphrodites and DWP70 hermaphrodites with *ife-3(ok191)* balanced by *nT1[qIs51]*. To ensure the *ife-3(ok191)* mog phenotype was fully penetrant, worms were maintained at 20°C. Demonstrating the efficacy of *fem-3(RNAi)*, we found that 10 to 47% of the progeny of four treated wild-type animals developed as females that lacked fertilized embryos and contained an excessive number of oocytes in their proximal gonad arms, both of which were apparent through a dissecting microscope. Similarly, for five *fem-3(RNAi)*-treated DWP70 animals, 50 to 80% of their GFP-positive heterozygous progeny appeared female when viewed using a dissecting microscope. Absence of embryos and excess of oocytes in the proximal gonad arms of these females was confirmed using DIC microscopy (compare Figs 2 and 3A to Fig. 5). We were unable to unambiguously identify GFP-negative *ife-3(ok191)* homozygous progeny that were female using a dissecting microscope due to the fact that these animals normally lack embryos and have small gonads. However, we examined nine randomly selected GFP-negative progeny using DIC microscopy, and found that seven contained oocytes and smaller oocyte-like cells in their proximal gonad arms, and six of these appeared to be true females that lacked fertilized embryos (Fig. 5). Thus, inhibition of *fem-3* bypasses the requirement for *ife-3* in promoting oogenesis, indicating *fem-3* is epistatic to *ife-3*.

## DISCUSSION

We demonstrate that the canonical eIF4E isoform of *C. elegans* encoded by *ife-3* is required for proper body growth, and is a novel regulator of sex-determination in the hermaphrodite germline. This differs from previous studies showing that RNAi-mediated knockdown of *ife-3* prevents embryonic morphogenesis (Keiper et al., 2000; Sönnichsen et al., 2005). This discrepancy is likely due to RNAi eliminating an essential maternal gene function and masking zygotic gene functions (Ahringer, 2006). Confirming this phenomenon for *ife-3*, we found that animals completely lacking *ife-3* due to a large genomic deletion are still able grow to adulthood so long as they are the progeny of heterozygous parents (Table 2; Fig. 1B). On rare occasions when *ife-3* homozygous mutants produce embryos, which receive no maternal *ife-3* activity, these embryos arrest before completion of morphogenesis, matching the effects of RNAi (Table 3). Thus, maternal but not zygotic *ife-3* is essential for embryogenesis.

Mutants for *ife-3* have stunted growth (Fig. 2, supplementary material Fig. S1A), a phenotype reasonably expected from loss of an eIF4E homolog that promotes protein synthesis. Also, *ife-3* mutant gonads are smaller and contain fewer germ cells than those of wild-type animals (Fig. 4A,B). A somewhat less intuitive effect of absence of *ife-3* is a disruption of hermaphrodite germline sex-determination. Germline sex-determination in *C. elegans* is regulated by successive layers of genes that alternatively promote spermatogenesis or oogenesis, with each layer inhibiting the activity of the layer immediately downstream (for review, see Ellis and Schedl, 2007). The final output of spermatogenesis or oogenesis depends on the balance of these successively opposing influences. In male *C. elegans* worms, defined genetically by a single X chromosome, *her-1* gene activity pushes this path toward spermatogenesis throughout their fertile life. In XX hermaphrodites, sex-determination is also initially tipped toward spermatogenesis during larval development, but somehow switches toward oogenesis in adulthood. We find that this switch normally requires *ife-3*, as XX *ife-3* mutants grown under standard conditions have a normal hermaphrodite soma but a masculinized germline (mog phenotype) that produces sperm and fails to produce oocytes (Figs 3 and 4A,C, supplementary material Fig. S1B,C).

Another key gene involved in the spermatogenesis-to-oogenesis transition is *fem-3*. Loss-of-function alleles of *fem-3* result in female XX worms that produce oocytes but never produce sperm, while gain-of-function alleles result in the XX worms with the mog phenotype (Hodgkin, 1986; Barton et al., 1987). Thus, *fem-3* promotes spermatogenesis during larval development, but its activity must be restrained to permit the transition to oogenesis in adulthood. Many genes that promote the spermatogenesis-to-oogenesis transition function genetically as upstream inhibitors of *fem-3*. Thus, while mutations in these genes cause a mog phenotype in XX animals, the additional loss of *fem-3* reverses this effect to produce XX females. Similarly, the mog phenotype of *ife-3* mutants is reversed by RNAi-mediated inhibition of *fem-3*. This suppression of the *ife-3* phenotype is specific to sex-determination, as *fem-3(RNAi)* does not restore normal body growth or gonad size to *ife-3* mutants, leaving small female worms with few, small oocytes (Fig. 5).

Inhibition of *fem-3* is at least in part through suppression of its translation. The *fem-3* 3′UTR bears a *cis-*acting point mutation element (PME) that when mutated, results in masculinizing gain-of-function alleles (Ahringer and Kimble, 1991; Barton et al., 1987). Attachment of the *fem-3* 3′UTR is sufficient to inhibit expression of exogenous reporter genes such as *lacZ*, while a *fem-3(gf)* 3′UTR with a mutated PME has no such effect (Gallegos et al., 1998). Fem-3 binding factor (FBF), encoded by *fbf-1* and *fbf-2*, promotes the spermatogenesis-to-oogenesis transition by binding to the PME *in vivo*. Consequently, *fbf-1/fbf-2* mutants have a mog phenotype, and wild-type FBF cannot bind the PME of mog-causing *fem-3(gf)* alleles (Zhang et al., 1997). Six additional genes, *mog-1*, *-2*, *-3*, *-4*, *-5*, and *-6*, are also important for PME-dependent inhibition of *fem-3*, as loss-of-function alleles result in a mog phenotype and also prevent PME-containing 3′UTRs from inhibiting expression of *lacZ* (Graham and Kimble, 1993; Graham et al., 1993; Gallegos et al., 1998). However, it is not clear how the products of the *mog* genes contribute to this inhibition.

Interestingly, the *mog* genes and *ife-3* share mutant phenotypes beyond the mog. *mog* mutants also have poor body growth and small gonads (Graham and Kimble, 1993; Graham et al., 1993). Also, when oogenesis is induced in *mog* mutants (e.g. through inhibition of *fem-3*) and resultant *mog* oocytes are fertilized, the progeny die as embryos or larvae, demonstrating maternal effect lethality. The MOG gene products share no similarity with IFE-3, but they are also involved in RNA metabolism, with *mog-1*, *-4*, and *-5* encoding homologs of mRNA splicing factors, *mog-2* encoding a U2 snRNP protein, *mog-3* encoding a small nucleolar preRNA-splicing protein, and *mog-6* encoding a divergent cyclophilin that associates with MEP-1, an RNA-binding protein also required for PME-mediated inhibition of *lacZ* (Belfiore et al., 2002, 2004; Kasturi et al., 2010; Puoti and Kimble, 1999, 2000; Zanetti et al., 2011). The *mog* genes and *ife-3* appear to be part of an even wider set of genes that are involved in RNA functions, and whose mutation results in poor growth, maternal effect lethality, and germline masculinization, including *ddx-23* encoding an mRNA splicing factor, *mag-1* encoding a *mago nashi* homolog, and *R07E14* encoding a homolog of Y-14 (Kawano et al., 2004; Konishi et al., 2008; Li et al., 2000). These similarities hint at the possibility of some commonality in the function of these genes.

As eIF4E proteins generally act to promote mRNA translation, it seems unlikely that *ife-3* participates directly in PME-mediated inhibition of *fem-3* translation, but may promote translation of some other factor that is more directly involved. Alternatively, eIF4E-dependent inhibition of translation can arise in response to eIF4E-binding proteins (4E-BPs), as demonstrated by the *Drosophila* 4E-BP, Bicoid. Association of Bicoid with the *caudal* mRNA 3′UTR inhibits translation when the mRNA is associated with the eIF4E isoform encoded by *d4EHP*, but not when it is associated with the isoform encoded by *deIF4E* (Cho et al., 2005). In light of this, it might be significant that mutation of 4E-BP-encoding *ifet-1* mildly masculinizes the *C. elegans* hermaphrodite germline (Sengupta et al., 2013). Unfortunately, we were unable to determine directly whether *ife-3* is required for PME-dependent inhibition using the *lacZ* reporter developed by Gallegos and colleagues (1998), as loss of *ife-3* inhibited expression of the reporter in a PME-independent manner (data not shown).

An intriguing alternative model for how *ife-3* might promote the spermatogenesis-to-oogenesis transition is provided by a suggestion by Goodwin and Ellis (2002). They suggest the tipping in the balance between spermatogenesis to oogenesis in the germline might be tied to growth of the germline in terms of cell size or number. Based on such a model, *ife-3* mutants might be defective in the spermatogenesis-to-oogenesis transition as a simple consequence of their poor body growth and small gonad size. Such a model might also help account for the recurrent pairing of poor body/gonad growth and mog phenotype among many sex-determination mutants.

An interesting question is why the other eIF4E isoforms cannot replace *ife-3* function. Two possible contributions are differential expression patterns, and differential specificity for mRNA 5′ caps. In terms of expression, IFE-3 is enriched in the germline over the soma (Amiri et al., 2001). Unfortunately, our transgene rescue experiments shed little light on where *ife-3* function is required, as we used the endogenous *ife-3* promoter to drive expression *ife-3* from a complex extrachromosomal array, which can support expression in the germline or the soma. However, maternal effect lethality and the mog phenotype often correlate with germline expression of the affected gene. We therefore might have expected suppression of these phenotypes by *ife-1*, *-2*, or *-5*, all of which have some function and/or enriched expression in the germline, but not *ife-4*, which is soma-enriched (Amiri et al., 2001; Dinkova et al., 2005; Song et al., 2010). However, the eIF4E isoforms encoded by *ife-1*, *-2*, and *-5* all differ from IFE-3 in their recognition of mRNA caps. In *C. elegans*, mRNAs acquire a 5′-5′ linked monomethylated guanosine (MMG) cap, but this is often replaced with a trimethylated guanosine (TMG) cap during the *trans-*splicing (Blumenthal, 2012). While IFE-3 and -4 bind MMG caps exclusively, IFE-1, -2, and -5 bind both cap structures (Jankowska-Anyszka et al., 1998; Keiper et al., 2000). Thus, TMG-capped mRNAs in the germline might prevent these isoforms from efficiently binding MMG-capped IFE-3 targets. Whether expression pattern and cap-specificity are sufficient to explain the unique roles of *ife-3*, or whether factors such as 4E-BPs contribute, remains to be determined.

## MATERIALS AND METHODS

### Plasmids, primers and molecular techniques

pCR2.1-*ife-3* was constructed by PCR amplification of genomic *ife-3*, including 2026 bp upstream and 386 bp downstream sequence, followed by TOPO-TA cloning (Thermo Fisher Scientific, Life Technologies, Grand Island, NY, USA). For production of double-stranded RNA (dsRNA) for *fem-3(RNAi)*, the *fem-3* coding region was PCR amplified from wild-type genomic DNA and TOPO-TA cloned. The *fem-3* sequence was then subcloned between two opposing T7 promoters in plasmid L4440 (Timmons and Fire, 1998), and dsRNA was synthesized *in vitro* with T7 polymerase using standard protocols. Supplementary material Table S3 lists all primers used in this study.

### Strains, growth conditions, and genetics

Worms were maintained under standard conditions at 20°C (Brenner, 1974), except where indicated. Strains N2 [*wild-type*] (Brenner, 1974), MT1083 [*egl-8(n488)* V] (Trent et al., 1983), and KX10 [*ife-3(ok191)/unc-34(e566)* V] were supplied by the Caenorhabditis Genetics Center (University of Minnesota, Minneapolis, MN, USA). FX02133 [*upsDf41 daam-1(tm2133)/+* V], the original source of *upsDf41* and *daam-1(tm2133)*, was provided by Shohei Mitani (National BioResource Project for the Experimental Animal Nematode *C. elegans*, Tokyo Women's Medical University School of Medicine, Tokyo, Japan).

The genomic rearrangement *nT1* behaves as a reciprocal translocation between ChrIV and ChrV (Edgeley et al., 2006). The variant *nT1[qIs51],* encoding pharyngeal GFP and a recessive lethal mutation, balances *ife-3(ok191)* in DWP70 [*+/nT1[qIs51]* IV; *ife-3(ok191)/nT1[aIs51]*V] and *upsDf41 daam-1(tm2133)* in XA8002 [*+/nT1[qIs51]* IV; *upsDf41 daam-1(tm2133)/nT1[qIs51]* V]. DWP72 [*+/nT1[qIs51]* IV; *upsDf41 daam-1(tm2133)/nT1[qIs51]* V; *upsEx40[ife-3]*] carrying the *ife-3*-containing extrachromosomal array *upsEx40*, was constructed through microinjection of XA8002 with pCR2.1-*ife-3* (25 ng/µl), mCherry marker plasmids (2.5 ng/µl pCJF90 [*Pmyo-2::mCherry*], 10 ng/µl pGH8 [*Prab-3::mCherry*], and 5 ng/µl pCJF104 [*Pmyo-3::mCherry*]) (Frøkjær-Jensen et al., 2008), and carrier DNA (75 ng/µl pRS315) (Sikorski and Hieter, 1989). RNAi was induced against *fem-3* by microinjection of *fem-3*-encoding dsRNA into the germline of young adult hermaphrodites (Wang and Barr, 2005). The F1 progeny resulting from eggs laid 24 h after injection were microscopically examined after 4 days growth.

To determine the viability of the progeny of *upsDf41 daam-1(tm2133)/+* heterozygous animals (Table 2), young adults were placed individually on plates with fresh bacterial lawns, and allowed to lay eggs for 4 h at 20°C. The adults were then removed, and the number of eggs counted on each plate. The following day (day 2), unhatched (dead) eggs were counted, larvae were counted on day 3, and adults were counted on day 4.

To assay for the ability of hermaphrodites to lay eggs (Table 3), L1 larvae were placed individually on plates with fresh bacterial lawns, and allowed to grow at the indicated temperatures. Each animal was examined for the presence of eggs or larvae each day of their fertile adulthood.

### Whole genome sequence analysis

Purified genomic DNA samples from N2 and XA8002 were submitted to the Cornell University Biotechnology Resource Center (Cornell University, Ithaca, NY, USA) for DNA library preparation and single-end 50 nucleotide sequencing (50× coverage) using Illumina HiSeq instrumentation. Illumina 1.8 datasets were uploaded to the Galaxy website (https://main.g2.bx.psu.edu/root), FASTQ groomed (Blankenberg et al., 2010), trimmed to remove 1 nucleotide from each sequence read end, and mapped as single-end reads against the *C. elegans* reference genome WS220 (http://www.wormbase.org) using Bowtie for Illumina (Langmead et al., 2009). To identify small nucleotide polymorphisms (SNPs), the resultant SAM format files were filtered to remove unmapped reads, and converted to a BAM format, from which a pileup was generated (Li et al., 2009). The pileup data were filtered to remove reads with quality lower than 20, and reads for genomic positions with sequence coverage lower than 3 (Li et al., 2009). SNPs present in ≥15% of reads at a given position were considered to represent potentially heterozygous or homozygous SNPs in XA8002 rather than sequencing errors. SNPs between ChrV coordinates 1 to 1,540,027, encompassing the sequence to the left of *daam-1* plus approximately 1 cM to its right, were considered *daam-1-*linked (supplementary material Table S1). To identify small deletions or insertions, FASTQ groomed files were mapped with BWA for Illumina (Li and Durbin, 2009), and searched using the Indel Analysis tool (supplementary material Table S1). To identify large genomic deletions or duplications, SAM datasets were converted to an interval format. Intervals that mapped to ChrV 1 to 154,000 were visualized against the *C. elegans* genome using the USCS genome browser (Genome Bioinformatics Group of University of California at Santa Cruz, Santa Cruz, CA, USA). Visually identified stretches over which the average number of sequence reads per position was approximately 50% lower than surrounding areas were considered possible heterozygous deletions. One region over which reads per position was increased >50% was considered to possibly indicate an amplified sequence.

### Staining and microscopic analysis

Live animals were visualized by differential interference contrast (DIC) microscopy as previously described (Mi-Mi et al., 2012). To visualize DNA, worms were fixed 4 min in fresh Carnoy's fixative (60% ethanol, 10% chloroform, 30% glacial acetic acid), rehydrated 3 min each in a 90%, 70%, 50%, and 25% ethanol series, washed in PBS, and stained 10 min in PBS with 1 µg/ml 4′6-diamidino-2-phenylindole (DAPI) before two final PBS washes. To stain for MSP, dissected worms were fixed 1 h in 100 mM potassium phosphate, pH 7.2 with 1.8% formaldehyde, washed with PBS/0.1% Tween-20 (PBS/T), post-fixed 5 min in −20°C methanol, and blocked 1 h in PBST with 1 mg/ml bovine serum albumin (PBS/TB) at room temperature. Incubations with MSP-specific antibody 4A5 (Kosinski et al., 2005) (Developmental Studies Hybridoma Bank, University of Iowa, Iowa City, IA, USA) at 1:200 dilution in PBS/TB, and Texas Red-labeled goat anti-mouse antibody (Rockland Immunochemicals, Limerick, PA, USA) at 1:8000 dilution in same, were each overnight at 4°C, with PBS/T washes between. DAPI (100 ng/ml) was included in the final wash.

Fluorescence and DIC images were acquired using a Cool-SNAP HQ2 digital monochrome charge-coupled device camera (Photometrics, Tuscon, AZ, USA) mounted on an Eclipse 90i upright research microscope (Nikon, Tokyo, Japan), and driven by NIS-Elements AR acquisition and analysis software (version 3.1; Nikon, Tokyo, Japan). Low magnification images (supplementary material Fig. S1A) were acquired using a DP-20 digital camera on an SZ61TR stereoimaging microscope, and driven by DP2-BSW software (Olympus, Center Valley, PA, USA). Images were processed linearly to enhance contrast using Photoshop CS4 (Adobe, San Jose, CA, USA).

### Statistical analysis

Quantitative data are presented as mean±one standard deviation. Results from experiments involving two data sets were subjected to unpaired, two-tailed Student's *t*-tests, with *P*≤0.05 considered statistically significant. Results from experiments involving three or more groups were subjected to one-factor analysis of variance followed by Fisher's least significant difference post hoc testing, with differences between groups exceeding the 95% confidence interval considered statistically significant.

## Supplementary Material

Supplementary Material
