# Supplementary material for: The canonical eIF4E isoform of C. elegans regulates growth, embryogenesis, and germline sex-determination
Source: Biol Open. 2015 May 15;4(7):843–51. doi: 10.1242/bio.011585 (PMC4571089; doi:10.1242/bio.011585)
Supplement: Supplementary Material [file supp_4_7_843__index.html]

The canonical eIF4E isoform of C. elegans regulates growth, embryogenesis, and germline sex-determination — The canonical eIF4E isoform of C. elegans regulates growth, embryogenesis, and germline sex-determination — Supplementary Material 

# The canonical eIF4E isoform of *C. elegans* regulates growth, embryogenesis, and germline sex-determination

## BIO011585 Supplementary Material

- Supplementary Material
