# Supplementary material for: The canonical eIF4E isoform of C. elegans regulates growth, embryogenesis, and germline sex-determination
Source: Biol Open. 2015 May 15;4(7):843–51. doi: 10.1242/bio.011585 (PMC4571089; doi:10.1242/bio.011585)
Supplement: Supplementary Material [file supp_bio.011585_BIO011585supp.pdf]

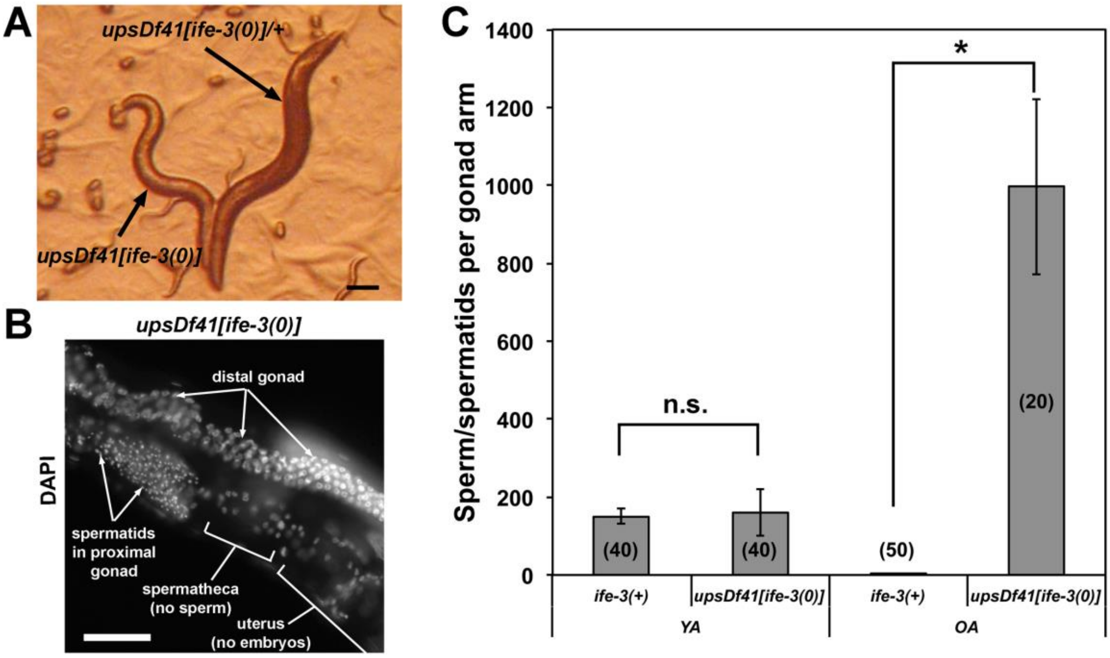

Fig. S1. Hermaphrodites homozygous for *upsDf41*, which deletes *ife-3*, exhibit poor growth and masculinized germlines. (A) A young adult XA8002 hermaphrodite that is heterozygous for *upsDf41* and *daam-1(tm2133)* (right) is larger than a young adult hermaphrodite that is homozygous for *upsDf41* and *daam-1(tm2133)* (left). Bar = 100 µm. (B) DAPI stain of a young adult *upsDf41* homozygous hermaphrodite shows immature germ cells in the distal gonad and spermatids in the proximal gonad, while the spermatheca is empty of mature sperm, and the uterus is empty of developing embryos. Bar = 50 µm. (C) Sperm/spermatid numbers decline from young adulthood (YA) to old adulthood (OA) in wild-type hermaphrodites, but increase in *upsDf41[ife-3(0)]* homozygous hermaphrodites. Results shown are typical of two independent experiments. The number of gonads analyzed per experiment is indicated in parentheses. Values are expressed as mean±one standard deviation. (*) indicates $p < 0.0001$. (n.s.) indicates no significant difference.

Table S1. SNPs and indels identified in XA8002

Position [a]	Gene affected [b]	Mutation characteristics [b]	Phenotypes of loss of gene function [c]
32,949	*egl-8*	point mutation in intron	egg-laying defect, constipated, aldicarb resistant, etc.
59,027	*cyp-29A3*	point mutation in upstream sequence	no reported phenotype
61,650	*cyp-29A3*	Δ 2 nt in coding sequence	no reported phenotype
166,566	*Y108G3AL.3*	Δ 1 nt in intron	no apparent phenotype
277,422	*npr-5*	Δ 1 nt in intron	lethal, sterile
311,489	*phf-10*	missense mutation in coding sequence	no apparent phenotype
312,639	*phf-10*	Δ 1 nt in upstream sequence	no apparent phenotype
363,817	ncRNA	point mutation	no apparent phenotype
626,135	*F48G8.7*	point mutation in intron	germline defects
658,179	*F31F4.17*	point mutation in upstream sequence	no apparent phenotype
	srh-24	point mutation in upstream sequence	no apparent phenotype
686,215	*F31F4.1*	point mutation in upstream sequence	no apparent phenotype

714,662	*srbc-40*	Δ 1 nt in down-stream sequence	no apparent phenotype
716,903	*srbc-40*	point mutation in intron	no apparent phenotype
854,920	*K09C6.9*	point mutation in intron	no apparent phenotype
1,000,028	*B0454.13* (ncRNA)	point mutation	no reported phenotype
	ergo-1	point mutation in upstream sequence	enhanced RNAi, increased mRNA levels
1,047,177	*nra-4* intron	point mutation in intron	levamisole and aldicarb resistant, embryonic lethality
	B0454.13 (ncRNA)	point mutation	no reported phenotype
1,256,994	*B0454.13* (ncRNA)	Δ 1 nt	no reported phenotype
1,260,909	*C39F7.1*	point mutation in intron	dumpy, paralyzed, slow growth
	B0454.13 (ncRNA)	point mutation	no reported phenotype
1,289,796	*egl-2*	silent mutation in coding sequence	egg-laying defect, flaccid, kinked
1,361,887	ncRNAs	point mutation	no reported phenotype
	snt-3	point mutation in	no apparent phenotype

		upstream sequence	
1,447,331	*cnc-4*	Δ 1 nt in intron	no apparent phenotype
1,474,607	ncRNAs	point mutation	no reported phenotype
	unc-60	point mutation in upstream sequence	embryonic lethal, thin, sluggish, etc.
1,540,027	*oac-30*	point mutation in intron	no apparent phenotype

[a] Positions are coordinates on ChrV according to www.wormbase.org, WS244.

[b] Predictions of genes affected are according to www.wormbase.org, WS244. Deletions (Δ) are followed by the number of nucleotides (nt) deleted.

[c] Phenotypes are those reported for mutations or RNAi-mediated knockdown of the indicated gene, according to www.wormbase.org, WS244. "No apparent phenotype" indicates analysis showed no defect, while "no reported phenotype" indicates no analysis has been performed.

Table S2. Putative large deletions or sequence amplifications near *daam-1* on ChrV in XA8002

Sequence variation [a]	Genes affected [b]	Phenotypes of loss of gene function [c]
Δ 300 - 54,610 (*upsDf41*)	*cTel3X.2*	no apparent phenotype after RNAi
	cTel3X.3	no apparent phenotype after RNAi
	cTel3X.1	no phenotypic data available
	B0348.5	no phenotypic data available
	ife-3	embryonic lethality after RNAi (Keiper et al., 2000; Sönnichsen et al., 2005)
	egl-8	egg-laying (Trent et al., 1983), defecation (Thomas, 1990) defects after mutation, some embryonic lethality after RNAi (Skop et al., 2004)
	B0348.7	no phenotypic data available
	B0348.8	no phenotypic data available
	B0348.2	no apparent phenotype after RNAi
	B0348.1	low-penetrance molting defect after RNAi
	srt-52[d]	low-penetrance molting defect after RNAi
ampl 54,610 - 62,500	*srt-52*[d]	low-penetrance molting defect after RNAi
	cyp-29A3	short defecation cycle after RNAi
Δ 66,800 - 67,600 (*tm2133*)	*daam-1*	no apparent phenotype from mutation or RNAi (this study)
Δ 75,400 -	*daam-1*	no apparent phenotype from mutation or RNAi (this

75,600	intron	study)
Δ 120,200 - 120,400	*cgp-1* intron	altered bacterial resistance after RNAi
Δ 152,200 - 152,600	upstream of *W03F9.4*	no apparent phenotype after RNAi

[a] Putative deletions (Δ) were inferred from regions of the genome for which we observed an approximately 50% smaller average number of reads per position compared to surrounding regions, as described in Materials and Methods and shown in Fig. 1A for *upsDf41*. One region shows a putative sequence amplification (ampl), with one or more additional copies present in XA8002 based on a >100% increase in the average number of reads per position in this region. Numbers indicate coordinates on ChrV according to www.wormbase.org, WS220.

[b] Genes located within putative genomic variations according to www.wormbase.org, WS220. All variations affect one or more exons of the indicated genes, except where noted.

[c] Phenotypes associated with mutations or RNAi-mediated knockdown, according to www.wormbase.org, WS244 or as cited.

[d] *srt-52* is partially overlaps both *upsDf41* and a neighboring putative amplification.

Table S3. Primers used in this study

Primer name	Purpose	Sequence
fhod-2+for	detect *daam-1(+)*	CTGGGAACAAATTTTTTGAAAATTTTAGATTCGCGCCA
fhod-2+rev	detect *daam-1(+)*	GGAACAAGTGCTTCAACTCTGTCATGG
fhod-2tm2133for	detect *daam-1(tm2133)*	GGAGATGTCGGGCCATGCATTTTAG
fhod-2tm2133rev	detect *daam-1(tm2133)*	GCTGCAGTTTCAGACTGGCTAAACC
egl-8testF	detect *egl-8*	CCATTGAATGTCTCCCATACAGGAAGC
egl-8testR	detect *egl-8*	CGTTTCAAGCAACCTGTCATTCTATTTATTCTTGC
ife-3ok191for	genotype *ife-3*	CTGTGCGAATAACGAGTTTTCAGCGA
ife-3ok191rev	genotype *ife-3*, analyze *ife-3* sequence	GACTGAATGTGATTGTACAGCGACC
ife-3F2	amplify genomic *ife-3*, analyze *ife-3* sequence	GGAGCGTTTTCAATAGCTCGTGGAG
ife-3R	amplify genomic *ife-3*, analyze *ife-3* sequence	CAAACGCCCTACAAATCATGTGTGTGAAG
ife-3cDNAfor	analyze *ife-3* sequence	ATGAGCACATCCGTAGCGGAAAACAAA
ife-3cDNArev	analyze cloned *ife-3*	TTAAGGAGTTGGGGTGGCTGGAG
fem-3RNAiF	clone *fem-3* for RNAi	GCGGCCGCATGGAGGTGGATCCGGGTTCAG
fem-3RNAiR	clone *fem-3* for RNAi	GCGGCCGCTCATCGTTTCCTGGAGCAATCAGTAGCA
